# Adult-Onset Cerebral Adrenoleukodystrophy Without Adrenal Gland Involvement

**DOI:** 10.7759/cureus.9813

**Published:** 2020-08-17

**Authors:** Aniruddh Mannari, Brandon Wiggins, Ghassan Bachuwa

**Affiliations:** 1 Internal Medicine, Hurley Medical Center, Michigan State University, Flint, USA

**Keywords:** adrenoleukodystrophy, neurogenetics, demylinating disorder, multiple sclerosis, neuroradiology, spinal cord, mutation, adrenal insufficiency, vlcfa, peroxisome

## Abstract

X-linked adrenoleukodystrophy (X-ALD) is a metabolic disorder characterized by endocrine and neurological degeneration. A rare and variegated entity in adults, diagnosis is often a significant challenge and can lead to extensive testing, including invasive procedures if clinical suspicion is not high. We present the case of a 46-year-old-male with neurological dysfunction that is uncommon in X-ALD with preserved adrenocortical function. Initially misdiagnosed with multiple sclerosis, the patient faced a significant neurological and cognitive decline in a short follow-up time frame, ultimately losing meaningful independent function. Emphasis is placed on criteria for appropriate discernment based on imaging studies and previous literature data. We also highlight the value of shared decision making to maximize the quality of life in such advanced stage neurodegenerative disorders.

## Introduction

X-linked adrenoleukodystrophy (X-ALD) is an inherited neurodegenerative disorder caused by a mutation of the ATP-binding cassette, subfamily D, member 1 (ABCD1) gene that encodes for the protein involved in the metabolic pathway of very long chain fatty acids (VLCFAs) in cellular peroxisomes [1]. Accumulation of VLCFAs causes demyelination in the central nervous system (CNS) and adrenal insufficiency in the adrenal cortex [2]. X-ALD occurs in approximately 1 in 20,000 births for hemizygotes and 1 in 16,800 for heterozygotes [3]. As clinical signs and symptoms overlap considerably with more common demyelinating, cerebrovascular, or metabolic disorders, diagnosis and disposition into an appropriate pipeline of care is a challenge without clearly defined guidelines. Unlike its childhood counterpart, adrenoleukodystrophy of adult onset is exceedingly rare, comprising 2-5% of total confirmed cases [4]. Previous observations suggest that adrenal insufficiency is absent in 20% of cases [5]. X-ALD is a part of the standard Recommended Uniform Screening Panel for state-mandated newborn screening in the United States [6]. Definitive diagnosis consists of genetic testing for the mutated ABCD1 gene in males and with carrier gene sequencing [7]. Curative interventions with hematopoietic stem cell transplant (HCT) in patients with milder symptoms at relatively early onset have been somewhat successful [8]. However, such evidence in the adult subset is sparse. This case highlights the value of differentiating adult-onset X-linked adrenoleukodystrophy from other common neurodegenerative disorders in this age group. The diagnostic strategy, key elements of social context, and the importance of shared decision making are emphasized.

## Case presentation

A 46-year-old male with a past medical history of controlled essential hypertension presented with progressively worsening difficulty walking. His symptoms began 13 years prior, at age 33, as jerking clonic movements of his ankles that interfered with driving. Gait disturbance worsened over time. This was associated with urinary urgency. He did not seek clinical evaluation at that time. Due to his symptoms, and after episodes of urinary incontinence, he was unable to continue his work at the automobile assembly plant. His family history is notable for a younger brother (now deceased) who had leukodystrophy and a nephew (son of maternal aunt) who died from leukodystrophy in childhood. 

Over the past 13 years, he never developed speech difficulty, dysphagia, or limb weakness. He had no decline in cognitive function until six weeks prior to presentation when his wife noted him to be intermittently forgetful and confused. He was referred to neurology and underwent magnetic resonance (MR) imaging of the brain, which was initially concerning for multiple sclerosis. He was seen in the outpatient neurology clinic for a routine follow-up appointment. At that time, he was noted to be acutely confused and was not answering questions appropriately. He had ataxic gait and difficulty producing and comprehending speech. Due to acute worsening, he was sent to the emergency department for immediate evaluation. 

Neurological exam revealed an agitated and confused male. Cranial nerve examination showed right-sided myokymia of the face. There was no dysarthria. Pupils were of equal diameter and symmetrically reactive to light. Visual fields were intact bilaterally and extraocular movements intact without nystagmus. Coordination testing revealed bilateral dysmetria on finger-to-nose testing but no tremor. Non-sustained ankle clonus was present bilaterally. Muscle tone showed Ashworth grade 3 spasticity in the quadriceps, hamstrings, and adductor muscle groups bilaterally. Ashworth grade 2 spasticity was present in the right wrist. Babinski sign was flexor. He had hyperreflexia (3+) of the bilateral upper and lower extremities. Hoffman sign was positive. He had a spastic gait without toe dragging or circumduction. He was able to stand and ambulate without assistance.

Evaluation

Investigations

Earlier magnetic resonance (MR) imaging of the brain showed evidence of demyelinating lesions in the cortical brain and regions of the brainstem and spinal cord. He had previously been given a diagnosis of multiple sclerosis but had not started any disease-modifying therapy. CT angiography (CTA) of the head and neck was obtained shortly after presentation to the emergency department. No significant stenosis or malformations of the intracranial and extracranial arterial system were identified. There was mild atherosclerotic vascular calcification in the carotid artery without stenosis.

**Figure 1:**
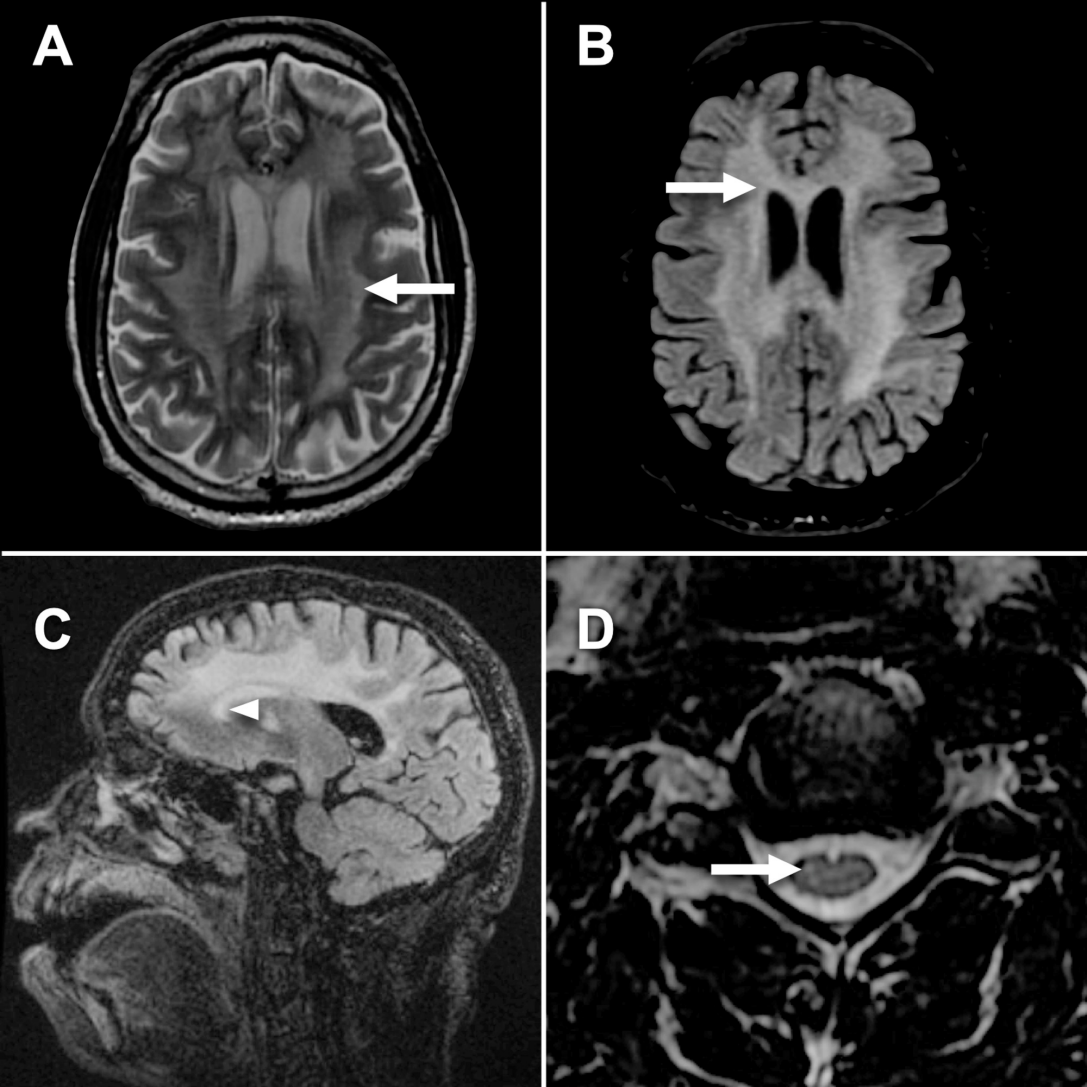
Multiplanar multisequence magnetic resonance (MR) imaging of brain and cervical spinal cord with and without gadolinium contrast. (A) T2 weighted sequence showing increased signal intensity in the white matter in a confluent distribution bilaterally (arrow). (B) T2 fluid attenuated inversion recovery (FLAIR) sequence with increased T2 signal involving same regions as T2 (arrow), but without meningeal signal increase. (C) Sagittal T2 FLAIR sequence showing involvement of the corpus callosum (arrowhead). (D) Axial T2 weighted sequence at the C5 spinal cord level showing small foci of increased T2 signal, suggesting regions of inflammation or demyelination (arrow).

Magnetic resonance (MR) imaging of the head, cervical spine, and thoracic spine were obtained to evaluate further causes of his mental status changes and physical examination findings. MR imaging showed bilateral regions of extensive, patchy, and confluent regions of increased T2 signal in the periventricular and subcortical white matter of the cerebral hemisphere in a bilateral distribution, including the corpus callosum (Figure 1). There was evidence of mildly increased T2 signal intensity of the C4 and C5 spinal cord levels. These findings are suggestive of demyelinating process but can also be caused by reactive inflammatory processes or infectious etiology.

Lumbar puncture was performed for further evaluation. Testing of cerebrospinal fluid (CSF) for cell culture, protein and cell profile, IgG level, and oligoclonal bands were negative or within normal limits. Additional testing of CSF for Venereal Disease Research Laboratory (VDRL), angiotensin-converting enzyme (ACE), paraneoplastic autoantibodies, Myelin basic protein, Human T-Lymphocyte Virus (HTLV) 1 and 2, Lyme serology, ceruloplasmin, heavy metal concentrations, and JC virus were negative or within normal limits.

Laboratory analysis showed no leukocytosis or anemia on complete blood count (CBC). Comprehensive metabolic panel (CMP) was within normal limits. Serum adrenocorticotropic hormone (ACTH) and cortisol levels were within normal limits. Peroxisomal fatty acid profile which evaluates presence of very long chain fatty acids (VLFCA) and branched chain fatty acids was obtained by Gas Chromatography-Mass Spectrometry (GC-MS) Stable Isotope Dilution Analysis at Mayo Clinic (Rochester, MN) to evaluate for suspected adrenoleukodystrophy. Results of relevant baseline laboratory investigations are provided in Table 1.

**Table 1:** Laboratory Evaluation WBC, white blood cell count. RBC, red blood cell count. IgG, immunoglobulin G. MBP, myelin basic protein. PCR, polymerase chain reaction; assay includes testing for *Haemophilus influenzae*, *Listeria* spp., *Neisseria meningitidis*, *Streptococcus agalactiae*, *Cytomegalovirus*, *Enterovirus* spp., Herpes Simplex Virus 1 and 2, Human Herpes Virus 6, and Varicella Zoster Virus. ACTH, adrenocorticotropic hormone. VLCFA, very large chain fatty acid. C22:0, C24:0 and C26:0 are saturated unbranched fatty acids with 22, 24 and 26 carbon atoms, respectively.

Assay	Result	Reference Range	Units
Cerebrospinal Fluid (CSF)			
Glucose	56	40-70	mg/dL
WBC	0	0 - <10	1/mm^3^
RBC	7	≤1	1/mm^3^
Clarity	Clear	Clear	
IgG	3.4	1.0 - 5.0	mg/dL
Albumin	20.8	8.0 - 35.0	mg/dL
MBP	<2.0	2.0 - 4.0	mg/dL
Viral profile, PCR	Nonreactive	Nonreactive	-
Oligoclonal band	Negative	Negative	-
Serum			
Cortisol, morning	12	4.3 - 22.4	mg/mL
ACTH	39	< 46	pg/mL
Peroxisomal VLCFA Profile (Serum)			
C24:0/C22:0	1.76	≤ 1.39	-
C26:0/C22:0	0.050	≤ 0.023	-
C24:0	50.0	≤ 96.3	nmol/mL
C22:0	88.1	≤ 91.4	nmol/mL
C26:0	2.52	≤ 1.30	nmol/mL
Phytanic Acid	0.61	≤ 9.88	nmol/mL
Pristanic Acid	0.05	≤ 2.98	nmol/mL
Pristanic:Phytanic Ratio	0.08	≤ 0.39	-

Diagnostic Strategy

The presence of diffuse patchy lesions involving the white matter of the brain must be carefully evaluated. Considering the temporal features of the patient’s symptoms and the imaging findings, a primary central nervous system disorder emerged as the most likely diagnosis. Multiple sclerosis was initially considered; however, his symptom onset was only a few weeks prior to presentation without any prior signs of hyperreflexia, ocular manifestations, or focal neurologic signs. Additionally, all the lesions on MRI appeared recent without demonstration of lesions distanced across space and time. CSF studies were unremarkable for a definitive etiology. Notably, oligoclonal banding, seen in multiple sclerosis (sensitivity 85-90%), resulted negatively [9]. Hence, an alternative diagnosis was sought. 

Vasculitis of the central nervous system was also considered in light of multi-territory involvement of the T2 changes in the white matter. However, CT angiogram showed no evidence of stenosis in the intracranial arterial circulation, and no signs of ischemia were evident on diffusion-weighted imaging (DWI). Even in cases with high suspicion, a definitive diagnosis can be challenging as even the digital subtraction angiography (DSA), and the gold standard arterial biopsy can be non-revealing for evidence of angiitis.

The patient’s family history of adrenoleukodystrophy is contributory. A peroxisome profile showed elevated levels of VLCFAs. There is presence of long-chain fatty acids of the C24:0/C22:0 and C26:0/C22:0 ratios, which suggests hemizygosity for X-linked adrenoleukodystrophy (X-ALD). Confirmation is often obtained with a positive mutation in the ABCD1 gene.

Other rare causes for this presentation include (1) cerebral autosomal dominant arteriopathy with subcortical infarcts and leukoencephalopathy (CADASIL) which was excluded due to absence of lacunar infarcts and punctate hemorrhage and (2) progressive multifocal leukoencephalopathy (PML) which is excluded based on the absence of JC Virus in the CSF [10]. 

Treatment and follow-up

Initially suspecting an acute exacerbation of multiple sclerosis, he was given a course of intravenous methylprednisolone 500 mg twice daily for five days. He was given divalproex and olanzapine which improved his behavior without side effects. However, his spastic gait did not improve despite steroid therapy. In order to evaluate adrenal function, serum cortisol and adrenocorticotropic hormone (ACTH) levels were obtained as seen in Table 1. Since these values were obtained after completing steroid therapy, these levels could potentially be falsely normal and have limited utility in this case. No prior adrenal function studies were available for comparison. He underwent physical and occupational therapy evaluation and continued outpatient physical therapy sessions to improve mobility. He was informed about the expectations of the clinical course of adrenoleukodystrophy and the supportive nature of its management. He agreed to continue follow-up with his neurologist within two weeks to obtain outpatient genetic testing for definitive diagnosis.

**Figure 2:**
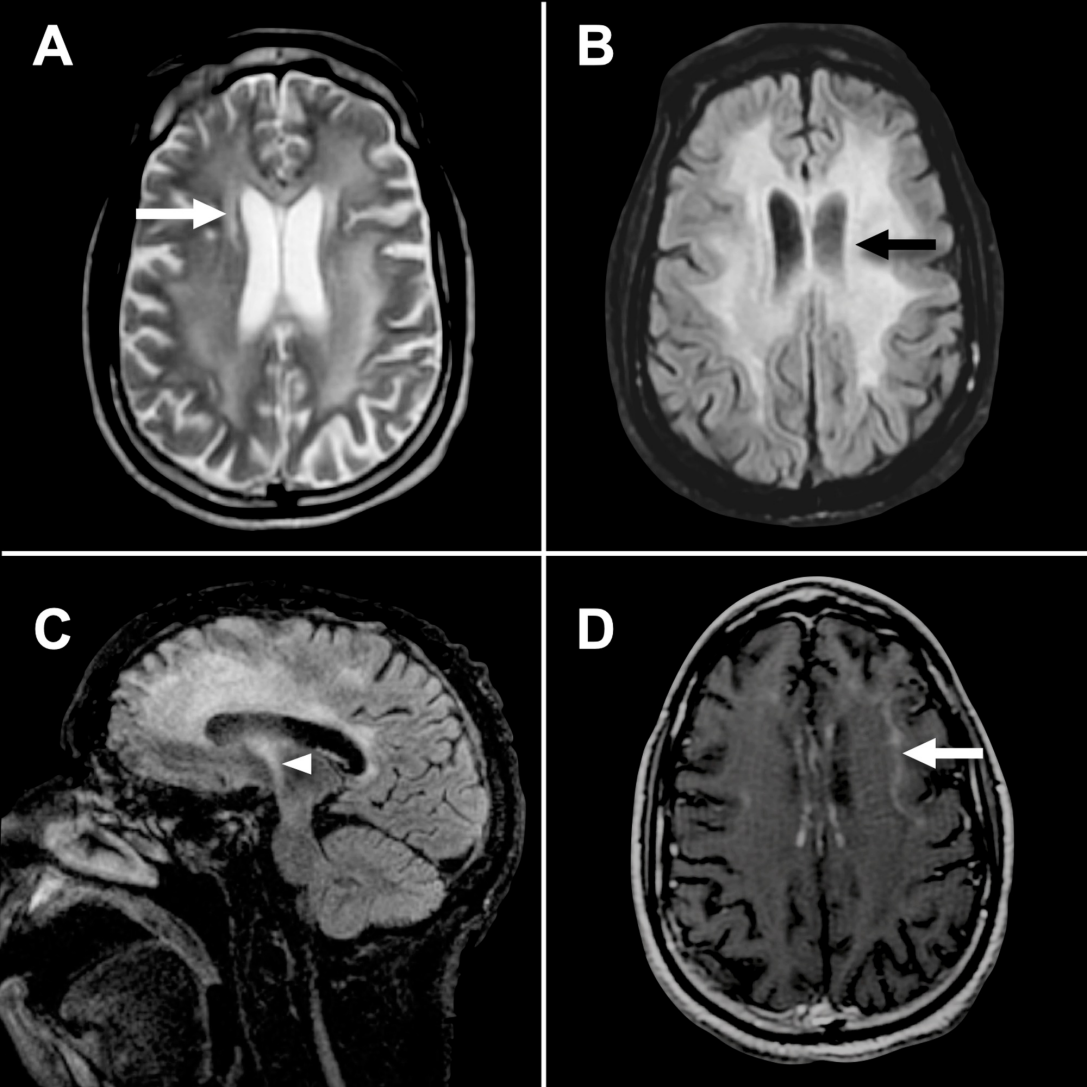
Follow-up multisequence magnetic resonance (MR) imaging of the brain with gadolinium contrast after a period of 60 days from initial MRI. (A) Axial T2 weighted sequence showing confluent areas of the T2 hyperintensity in the white matter bilaterally, with additional deep white matter involvement on follow-up (arrow). (B) T2 FLAIR sequence showing T2 hyperintense changes in the periventricular region without improvement compared to the prior study (arrow). (C) Sagittal T2 FLAIR sequence shows new regions of T2 hyperintense signal extending inferiorly along the corticospinal tract (arrowhead), predominantly on the left. (D) Axial T1 weighted post-contrast sequence demonstrating linear region of peripheral contrast enhancement (arrow).

After starting the patient on mood-stabilizing therapy and discussing follow-up plans, he was discharged home. He agreed to genetic testing for the ABCD1 gene mutation. The patient later sought the opinion of another neurologist at a local, tertiary care center in a metabolic/genetic neurology clinic. His mental status improved slightly, but he continued to demonstrate slow verbal response. He had no new focal deficits. His neurological exam was no different compared to that from his recent admission. He did not receive genetic testing for the ABCD1 gene mutation at that time because repeat imaging to evaluate the extent of demyelination was deemed to be of greater clinical significance. He underwent a follow-up MRI brain with gadolinium contrast. This revealed, in addition to previous findings seen in Figure 1, new regions of peripheral linear contrast enhancement predominantly in the left cerebral cortex suggesting active inflammatory demyelination (Figure 2).

Approximately two months following his initial admission, the patient developed increasing dysphagia and worsening aphasic speech. No new imaging studies were obtained at that time. He was evaluated by a gastroenterologist and underwent a percutaneous endoscopic gastrostomy tube placement and now requires continuous skilled nursing care. He cannot perform activities of daily living independently. Currently, the patient is under evaluation to consider bone marrow transplant as a compassionate therapy. Shared decision making and the establishment of goals of care and palliative options with the family are important in ensuring patient dignity and quality of life. Family members are often significantly burdened with grasping the gravity of such rapid progression. Close follow-up and establishing rapport with the family ensure the patient receives appropriate supportive care with the poor prognosis in mind.

## Discussion

The incidence of all phenotypes, including female carriers of X-ALD, is estimated to be 1 in 20,000-50,000 live births with the prevalence of adult-onset phenotype with cerebral involvement less than 3% [11]. According to contemporary literature, the phenotypes can vary greatly, ranging from rapidly progressive motor dysfunction leading to mortality during childhood to patients presenting with new onset of symptoms in adulthood. Genotype and phenotype in X-ALD are poorly linked, and prior investigations suggest that the mutation may act as a susceptibility factor with possibly contributory epigenetic and environmental factors. These mechanisms are poorly understood and could trigger inflammatory demyelination [4].

Adult-onset phenotypes of X-linked adrenoleukodystrophy, with its variable presentations, often pose a diagnostic challenge. In a previously reported case of a 21-year-old male, the presenting symptoms include auditory hallucinations, delusions, and seizure [12]. In another case, the diagnosis was made only after new onset of vision and speech impairment in a 43-year-old male following chemotherapy for multiple myeloma. No association between the specific chemotherapy agents and the development of symptoms or imaging changes was elucidated [13]. There is also variability of initial symptoms and age of onset. For example, in a case reported in Norway, a 61-year-old male was initially evaluated for Parkinson’s disease, who presented with additional signs of myelopathy. Later investigations revealed a diagnosis of X-linked adrenoleukodystrophy without any contributory family history. Phenotypic variation can be great in adult-onset subtypes of X-ALD [14].

Distinguishing X-linked adrenoleukodystrophy from multiple sclerosis

The patient in this case was initially suspected of having developed multiple sclerosis and was admitted to the hospital due to a concern for CNS vasculitis based on new cognitive deficits and worsening ambulation. However, a more detailed evaluation revealed cerebral adrenoleukodystrophy to be the most likely etiology. Notably, the patient did not have any clinical signs or laboratory evidence of adrenal insufficiency. However, this testing was performed after he completed the high dose methylprednisolone regimen which can potentially affect the accuracy of serum cortisol and ACTH levels.

Distinguishing multiple sclerosis from its common mimics, therefore, is a concept that is well-advised in contemporary literature and is valuable in this case [10]. As many patient presentations have an overlap of clinical symptoms and signs, imaging can often be used as a valuable tool for discernment. For instance, demyelination involving the posterior limb of the internal capsule and cerebral peduncles has been described in patients with an adult-onset subtype of X-ALD, an entity termed adrenomyeloneuropathy (AMN) [15]. While AMN has typically been described as having predominantly spinal cord and brainstem lesions, recent observations indicate that cerebral demyelination may be present in as high as 63% of affected patients according to a prospective observational study of 27 men with the AMN subtype of X-ALD [16]. 

Patient monitoring with surveillance imaging is important, particularly in those with worsening clinical symptoms or signs on neurological examination. MR imaging plays an important role as seen in the case of this patient. Follow-up imaging revealed regions of contrast enhancement in the left peripheral cortical brain. This commonly heralds the presence of active inflammatory disease activity contributing to his worsening neurological function. Current reports advocate for the value of contrast MR imaging with gadolinium. Notably, there is a documented association between the severity of contrast enhancement on T1-weighted MRI and the clinical progression of X-ALD [17].

The fact that the patient did not undergo genetic testing for the ABCD1 gene mutation for definitive confirmation is a limitation indeed, and it was not feasible in part due to his relatively progressive clinical course. Elevated VLCFA profile, clinical history, contributory family history, and imaging characteristics makes X-linked adrenoleukodystrophy the most likely diagnosis.

Review of current guidelines and novel therapies

While the childhood form has been studied in relatively greater detail and with larger sample sizes, adult-onset X-ALD treatment is often limited to supportive and palliative care due to the progression of deficits. Hematopoietic stem cell transplant (HCT) is a compassionate use option currently being investigated. A retrospective study of patients with an adult-onset cerebral phenotype of X-ALD who underwent HCT as a palliative measure showed a survival rate of 57% during the follow-up period [18]. These data indicate that the patient in this case likely has a greater chance of harm than benefit if he underwent HCT. An early-stage gene therapy described by Gong et al. showed the successful expression of vector inserted ABCD1 gene in vitro. Consequently, measurements of VLCFA within peroxisomes was markedly decreased in cell lines of X-ALD treated with gene insertion in culture [19]. A human gene therapy trial in the childhood form of X-ALD showed promising early data and test subjects who received transfusion of CD34+ cells with vector insertion of ABCD1 gene showed decreased rates of progression of cerebral demyelination and neurological decline compared to controls [20]. These studies show the importance of the possibility of targeted gene therapy as a viable an alternative to HCT, especially in cases where profound neurological damage has not yet manifested.

## Conclusions

Adult-onset X-linked adrenoleukodystrophy is a rare entity which poses a diagnostic challenge due to its clinical variability. This case of a middle-aged male with new-onset upper motor neuron symptoms and rapid course highlights the uncommon presentation of this disorder, particularly in non-carriers. Although X-ALD is included in modern newborn screenings, delayed onset of symptom manifestation can occur, and patients might not seek evaluation when asymptomatic. Hence, establishing clinical guidelines to evaluate patients with a thorough family history and broad differential is prudent. As seen in this case, the patient suffered a significant decline in physical and cognitive ability due to progressive inflammatory cerebral demyelination. This emphasizes the variable course of the disease and sheds light on the need for patient-centered shared decision-making about managing expectations of care, evaluating the risk and benefits of experimental treatments, and maintaining appropriate rapport and follow-up to maintain the highest quality of life possible.
